# Progress in Fevers

**Published:** 1903-12-19

**Authors:** 


*(To be continued.)*

### Progress in Fevers.

**Scarlet Fever.**—G. H. Weaver,[1] of Chicago, has made a study of the vitality of the organisms found in the throats of scarlet fever patients, and has arrived at the following conclusions:—(1) Streptococci are almost invariably present in the throat, being most abundant early in the disease; (2) they are as resistant as the other bacteria present and have resisted drying for 90 days; (3) they remain alive for a long time in milk; (4) scarlatinal streptococci do not appear to differ in cultural and morphological characters from streptococci from other sources. D. Forbes[2] points to the frequency of diphtherial infection in post-scarlatinal ear discharges and to the importance of detection and isolation in these cases. Further, he states that infection takes place by way of the Eustachian tube; that patients so infected show no clinical signs of diphtheria and appear immune to subsequent faucial or laryngeal infection; and that antitoxin has neither a prophylactic nor a curative action in such cases. J. W. Class,[3] of Chicago, has announced the discovery of the specific cause of scarlet fever in the shape of a giant diplococcus isolated from certain cases which occurred during an epidemic in the neighbourhood. Moser's scarlatina streptococcal serum has been tried by Hospischill[4] in 26 cases. Nine out of 12 children injected, in whom the prognosis was "doubtful but tending to be bad," recovered, and five out of 13 cases in which a fatal prognosis had been given also recovered. Fall of temperature and of pulse and respiration rate, improved mental condition, quiet sleep and greater inclination for nourishment were among the improvements observed. As pointed out by Fischer,[5] streptococci are so constantly found in association with scarlet fever that they appear to be a distinct etiological factor. Aronson maintains that the use of antistreptococcal serum should be continued as long as streptococci are found in the blood. He finds that virulent streptococci will grow and multiply in even powerful antistreptococcal serum, and considers that the curative properties of the latter are due to the development of anti-bodies, the "amboceptors" of Ehrlich, which prevent a general infection.

**Dysentery.**—The report of the Commission[6] appointed by the Secretary of State for War to investigate the occurrence of dysentery in South Africa and its relation to enteric fever has been published. The Commission consisted of Colonel Bruce, who was in charge of the laboratory work, and Colonel Notter and Dr. Simpson, to whom was allotted the inspection of camps, water supply, etc. The results of Bruce's pathological investigations were mainly negative, for he found no relationship between dysentery and amœbæ or any species of micro-organism hitherto regarded as causative of dysentery, and none between dysentery and enteric fever. In the second part of the report the general history of the outbreaks of both diseases is fully detailed by Simpson. During the first and second years there were 24,274 admissions for dysentery with a mortality of 923, or 4 per cent., and 31,118 admissions for enteric with a mortality of 6,177 or 19·7 per cent. In each case the mortality was lower in the second year. The general insanitary nature of the environment as regards drainage, soil, disposal of excreta, insufficient protection of food, flies, and, above all, polluted surface and river water supply, are regarded as furnishing ample sources for the spread of these diseases. The comparative absence of fatal dysentery, the usual scourge of armies in the field, is ascribed to the good supplies of perfectly fresh and well-cooked food. The lack of any specially appointed sanitary officer to carry out the recommendations made by the medical officer to the commanding officer is emphasised, and the suggestion made that the Royal Army Medical Corps, entirely exonerated from blame in this matter, should in the future consist of a medical branch and a health branch, and that the latter branch should include a few selected specially trained officers of the Royal Engineers. From this recommendation, as from certain other opinions expressed by Dr. Simpson, Colonel Notter dissents. It is calculated that the sickness due to enteric alone during the war cost the country between £3,500,000 and £4,000,000.

**Dengue Fever.**—A report has been issued concerning the study of the etiology of this disease in Manila,[7] where the disease is very prevalent. A brief review of earlier work in this field of investigation is given. In 1885, McLaughlin, studying the disease in Texas, isolated from the blood of patients a micrococcus which showed peculiar grouping when grown on artificial media. In 1897, Gamon, in another epidemic in Texas, found a different coccus, allied, he thought, to one of the pus organisms. In 1902, Graham, investigating an epidemic in Syria, found in the blood of each of the 100 cases he studied a hæmatozoon somewhat resembling the plasmodium malariæ, occupying the red corpuscles, but unpigmented and exhibiting so long a cycle of reproduction that it was difficult to follow. It was too dull in colour to be mistaken for a vacuole. The conclusions reached from a study of the Manila epidemic are :—(1) In dengue fever there is no leucocytosis ; (2) the white corpuscles are present in normal proportions ; (3) neither Graham's hæmatozoon nor McLaughlin's micrococcus has been found ; (4) the etiological factor of the disease is as yet unknown. Graham's claim that the disease is disseminated by mosquitoes is being investigated.

[1] Med. Rec., 1903, July 11. [2] Brit. Med. Jour., June 13, p. 1393. [3] Med. Rec., Aug. 22, p. 292. [4] Edinburgh Med. Jour., July. [5] The Therapist, May 15. [6] Lancet, Aug. 29. [7] Med. Rec., May 2.

*(To be continued.)*

